# A Novel Index in Hepatocellular Carcinoma Patients After Curative Hepatectomy: Albumin to Gamma-Glutamyltransferase Ratio (AGR)

**DOI:** 10.3389/fonc.2019.00817

**Published:** 2019-09-04

**Authors:** Junyi Shen, Li Tang, Xiaoyun Zhang, Wei Peng, Tianfu Wen, Chuan Li, Jiayin Yang, Guanjian Liu

**Affiliations:** ^1^Department of Liver Surgery and Liver Transplantation Center, West China Hospital, Chengdu, China; ^2^Intensive Care Unit (ICU), West China Hospital, Chengdu, China; ^3^Department of Evidence-Based Medicine and Clinical Epidemiology, West China Hospital, Chengdu, China

**Keywords:** albumin to gamma-glutamyltransferase ratio, platelet to lymphocyte ratio, prognosis, hepatocellular carcinoma, hepatectomy

## Abstract

**Aim:** As high gamma-glutamyltransferase level or low albumin had negative impacts on the prognosis of hepatocellular carcinoma (HCC), the prognostic role of albumin to gamma-glutamyltransferase ratio (AGR) in HCC patients after hepatectomy remains unclear.

**Methods:** Between January 2007 and December 2015, 1143 HCC patients after hepatectomy were reviewed from a prospectively maintained database in West China Hospital. All qualified patients (*n* = 959) were classified as training set (year 2007–2012, *n* = 480) and validation set (year 2012–2017, *n* = 479). A time-dependent receiver operating characteristic (ROC) curve analysis was performed to evaluate the performance.

**Result:** AGR = 0.5 was identified as the best cut-off point to predict recurrence free survival (RFS) and overall survival (OS) in the training set. Low AGR was related to poor tumor characteristics and high systemic inflammation. Based on the multivariate analysis, high AGR was an independent predictor for better RFS and OS with an hazard ratio of 0.696 and 0.673. The high AGR group had better RFS and OS than the low AGR group in the training set as well as the validation set. The AGR-based score (AGR-PLR) could stratify HCC patients into three subgroups with different prognosis in the training and validation set. Patients with score 1 had a worse prognosis than those with AGR-PLR score 0, but better than those with AGR-PLR score 2. The predictive accuracy of the AGR-PLR score appeared superior to that of the AGR or PLR alone.

**Conclusions:** we firstly reported that AGR ≤ 0.5 was an independently prognostic factor in HCC after hepatectomy. The AGR-PLR score could further improve the discriminatory ability of prognosis.

## Introduction

Hepatocellular carcinoma (HCC) is one of most common malignancies worldwide, with an increasing incidence rate. Particularly, China accounts for 51% of the deaths from liver cancer worldwide because of the high prevalence of hepatitis B viral (HBV) infection (1, 2). Curative resection is widely accepted as an optimal therapy for patients with well-preserved liver function (3, 4). Unfortunately, the long-term survival for patients with HCC after hepatectomy is unsatisfactory. Cumulative evidences suggested inflammation and immunity contributed to tumor development and metastasis, severely affecting the patients' prognosis (5, 6). In recent years, some inflammatory scores like neutrophil-to-lymphocyte ratio (NLR) and platelet-to-lymphocyte ratio (PLR) represented the host immune response and were investigated for their prognostic role among patients with HCC. The scores could stratify the individuals with distinctive prognoses and were helpful for guiding the postoperative treatment (7–9).

Increasing evidences indicated elevated serum gamma-glutamyltransferase (GGT), an enzyme locating at the surface of the epithelial cells, which was related to inflammation and carcinogenesis. Abnormal GGT was a prognostic predictor for poor overall survival (OS) of HCC patients after transcatheter arterial chemoembolization (TACE) (10) or radiofrequency ablation (11). Albumin is a common indicator for malnutrition and liver dysfunction. Previous studies showed that lower serum albumin level or albumin-based markers were independent predictors of poor survival in several cancers (12, 13). High albumin to gamma-glutamyltransferase ratio (AGR) might aid the distinguishing between different prognoses of tumors. Zhang et al. proposed that AGR could predict the prognosis of intrahepatic cholangiocarcinoma (ICC) patients with a cut off value of 0.6 (14). In contrast to ICC, HCC commonly occurs with underlying liver disease, or even liver cirrhosis due to the high presence of viral hepatitis. The serum GGT and ALB are important index to evaluate inflammation and liver function. The role of AGR in HCC patients undergoing hepatectomy remains unknown.

In this study, we aimed to assess the prognostic role of AGR in HCC patients after radical hepatectomy. Furthermore, we would compare the predictive power between the novel AGR and other inflammatory scores for HCC patients after hepatectomy.

## Materials and Methods

**Figure 1:**
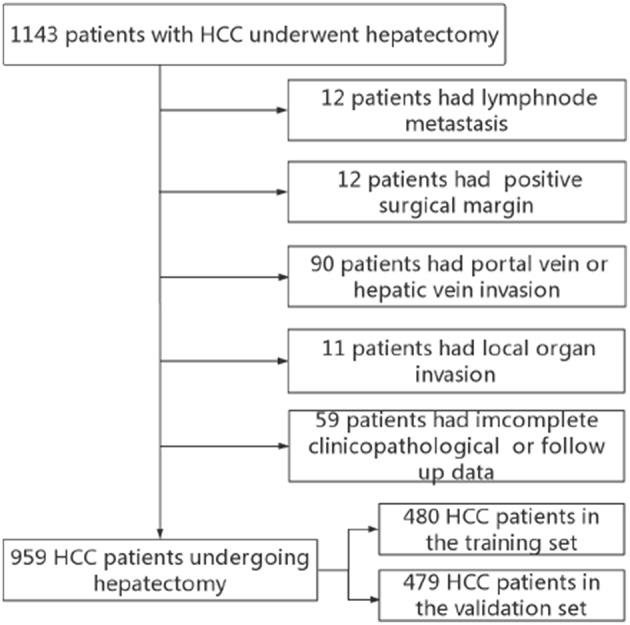
Flow chart for patient enrollment in current study.

Between January 2007 and December 2014, the data of 1,143 HCC patients who underwent hepatectomy were collected from the Department of Liver Surgery and Liver Transplantation Center in West China Hospital. Our inclusion criteria were as follows: (1) pathologically proven HCC; (2) first hepatectomy; (3) Child–Pugh grade A or Child–Pugh B. Exclusion criteria: (1) with major vascular invasion; (2) with positive surgical margin; (3) patients with recurrent HCC; (4) incomplete clinicopathological information or follow-up data. Finally, 959 patients were included in the current study. Patients between June 2007 and October 2012 were defined as the training set and patients between November 2012 to March 2015 were defined as the validation set (Figure 1). The following data were preoperatively collected: routine blood tests, liver function tests, the status of HBV infection, HBV-DNA load, alpha fetoprotein (AFP), tumor size, and number. Tumor differentiation, microvascular invasion (MVI) and satellite lesions were identified from pathological report. Liver cirrhosis was defined by Ishak score 5 or 6. The AGR is defined as preoperative serum albumin level divided by serum gamma-glutamyltransferase level. The study protocol conforms to the ethical guidelines of the 1975 Declaration of Helsinki and was approved by the ethics committee of West China Hospital, and written informed consent forms were obtained from all of the participants.

### Statistical Analysis

Frequency was compared between groups using the χ^2^ test with the Yates correction or Fisher's exact test. Continuous variables (presented as the mean ± SD) were compared using analysis of Student's *t*-test or the non-parametric Mann-Whitney test (data with non-normal distribution). The optimal cutoff value of AGR, PLR and NLR was determined by X-tile software (https://x-tile.software.informer.com/). The risk factors associated with prognosis of patients with HCC were evaluated using Cox regression analysis. Kaplan–Meier analysis and the log-rank test were performed by comparing the differences of the cumulative survival of HCC patients between groups. The discriminatory ability of AGR and PLR were evaluated by time-dependent area under receiver operating characteristic curve (AUROC). Time-dependent ROC was depicted using Kaplan-Meier method via the survival ROC package in R. The statistical analyses were performed using the SPSS 20.0 software (SPSS, Chicago, IL, USA) and R project version 3.4.1. A *P*-value <0.05 in two-tailed tests was considered as statistically significant.

## Result

### The Relationship Between AGR and Clinicopathological Features

**Figure 2:**
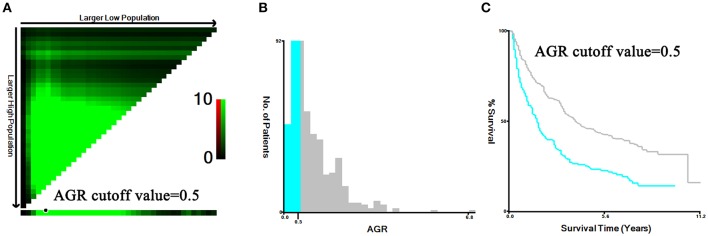
Identification for the cutoff points produced by X-tile plot in the training set. The prognostic power was strongest when the cutoff value of AGR was 0.5 **(A–C)**.

A total of 959 patients enrolled in our study were separated into the training set (*n* = 480) and the validation set (*n* = 479). In the training set, we adopted X-tile software to confirm that the optimal cutoff point for AGR was 0.5 by minimum *P*-value from log-rank χ^2^ test, based on which patients were divided into two groups with the strongest discriminatory capacity (Figure 2). Similarly, the optimal cutoff point for PLR was 167.7, and the optimal cutoff point for NLR was 3.1 (Figure S1).

Table 1 showed the demographic, clinical, and pathological features at baseline for both groups. There were 160 patients in the group A (AGR ≤ 0.5) and 320 patients in the group B (AGR > 0.5). Compared with group B, group A presented with: larger tumor sizes (*P* < 0.001); higher rate of patients with multiple tumors (*P* = 0.011); higher rate of the presence of MVI (*P* = 0.004); and satellite lesions (*P* = 0.001); higher Child-Pugh grade (*P* = 0.013); elevated ALT and AST (*P* < 0.001); higher NLR and PLR level (*p* = 0.036 and *p* = 0.013); and higher INR and Fibrinogen (*p* = 0.025 and *p* < 0.001). There were no significant differences about other variables between both groups (Table 1). The clinicopathological features of patients in the validation set was shown in the Table S1.

**Table 1:** Patient clinical characteristics between low and high AGR group in training set.

		**Low AGR group *n* = 160**	**High AGR group *n* = 320**	***p*-value**
Age	>60 years	37 (23.1)	83 (25.9)	0.576
Gender	Male	139 (86.9)	269 (84.1)	0.498
Positive HBsAg		141 (88.1)	280 (87.5)	0.884
Positive HBeAg		33 (20.6)	44 (13.8)	0.064
Positive HBcAb		156 (97.5)	315 (98.4)	0.489
Liver cirrhosis		107 (66.9)	207 (64.7)	0.684
Child-Pugh				0.013
	5	142 (88.8)	307 (95.9)	
	6	17 (10.6)	11 (34.0)	
	7	1 (0.6)	1 (0.3)	
	8	0 (0)	1 (0.3)	
Tumor size (cm)		7.2 ± 3.5	4.9 ± 2.5	<0.001
Tumor number				0.011
	One	128 (80.0)	281 (87.8)	
	Two	16 (10.0)	28 (8.8)	
	More	16 (10.0)	11 (3.4)	
BCLC	A	45 (28.1)	203 (63.4)	<0.001
	B	115 (71.9)	117 (36.6)	
MVI		54 (33.8)	68 (21.2)	0.004
Satellite lesions		24 (15.0)	19 (5.9)	0.001
Differentiation				0.741
	Poor	96 (60.0)	197 (61.6)	
	Moderate-well	64 (40.0)	123 (38.4)	
AFP	>400 ng/ml	69 (43.1)	116 (36.2)	0.164
TBIL (umol/L)		15.1 ± 5.6	15.2 ± 6.3	0.801
ALT (IU/L)		60.5 ± 46.6	42.4 ± 37.9	<0.001
AST (IU/L)		62.1 ± 34.2	38.4 ± 21.8	<0.001
ALB (g/L)		40.6 ± 4.0	42.4 ± 3.9	<0.001
GGT (IU/L)		178.1 ± 153.0	40.0 ± 18.7	<0.001
CREA (umol/L)		76.9 ± 15.2	79.4 ± 21.3	0.196
PLT (10 ∧ 9/L)		144.2 ± 75.2	131.3 ± 60.3	0.061
Neutrophil count		3.5 ± 1.5	3.3 ± 1.4	0.079
Lymphocyte count		1.5 ± 0.6	1.5 ± 0.5	0.516
INR		1.1 ± 0.1	1.1 ± 0.1	0.025
Fibrinogen		3.2 ± 1.1	2.7 ± 0.8	<0.001
PLR	>167.7	73 (45.6)	108 (33.8)	0.013
NLR	>3.1	71 (44.4)	110 (34.4)	0.036
Re-treatment				
	LT	2 (1.2)	6 (1.9)	
	Resection	9 (5.6)	28 (8.8)	
	Resection+TACE	5 (3.1)	11 (3.4)	
	RFA	14 (8.8)	20 (6.2)	
	RFA + TACE	3 (1.9)	15 (4.7)	
	Resection + RFA	3 (1.9)	3 (0.9)	
	Resection + RFA + TACE	1 (0.6)	6 (1.9)	
	TACE + Sorafenib	0 (0)	2 (0.4)	
	TACE	47 (29.4)	76 (23.8)	
	Sorafenib	2 (1.2)	0 (0)	
	BSC	44 (27.5)	37 (11.6)	

*AGR, albumin to gamma-glutamyltransferase ratio; HBsAg, hepatitis B virus surface antigen; HBeAg, hepatitis B virus e antigen; HBcAb, hepatitis B viral core antibody; MVI, microvascular invasion; AFP, alpha-fetoprotein,TBIL, total bilirubin; ALT, alanine transferase; AST, Aspartate aminotransferase; ALB, albumin; GGT, gamma-glutamyltransferase; CREA, Creatine; PLT, platelet; INR, international normalized ratio; PLR, platelet to lymphocyte ratio; NLR, neutrophil to lymphocyte ratio; LT, liver transplantation; TACE, transarterial chemoembolization; RFA, radiofrequency ablation; BSC, best supportive care*.

### AGR Related to Decreased Risk of Prognosis

The median RFS time of the training set was 64.5 months, with postoperative 1-, 3-, and 5-year RFS rates of 72.7, 48.6, and 36.7%, respectively. The 1-, 3-, and 5-year OS rates were 90.8, 70.3, and 56.0%, respectively, with a median survival of 86.0 months.

Based on the results of Univariate analysis, positive HBeAg, tumor size, tumor number, MVI, satellite lesions, elevated serum AST level, PLR, NLR, and AGR were significant factors affecting RFS (*p* < 0.05). In order to reduce the possible significant interactions among them, we selected significant variables in the univariate analysis and included them into the Cox proportional hazards model. We found that positive HBeAg (HR = 1.420, 95% CI 1.070–1.884, *p* = 0.015), tumor number(HR = 1.564, 95% CI 1.287–1.901, *p* < 0.001), MVI (HR = 1.562, 95% CI 1.285–2.096, *p* = 0.001), satellite lesions(HR = 1.817, 95% CI 1.290–2.560, *p* = 0.001), and PLR (>167.7: HR = 1.569, 95% CI 1.118–2.202, *p* = 0.009) were independent risk factors for RFS, while a higher AGR (>0.5: HR = 0.696, 95% CI 0.551–0.879, *p* = 0.002) was associated with decreased risk of RFS (Table 2).

**Table 2:** Univariate and multivariate analysis of factors related with recurrence free survival in training set.

	**HR**	***P***	**95% CI**	**HR**	***P***	**95% CI**
Age (>60)	0.945	0.653	0.738–1.210			
Gender	1.319	0.079	0.968–1.799			
Positive HBsAg	1.188	0.329	0.841–1.680			
Positive HBeAg	**1.386**	**0.021**	**1.049–1.830**	**1.420**	**0.015**	**1.070–1.884**
Positive HBcAb	0.893	0.767	0.422–1.889			
Liver cirrhosis	1.220	0.091	0.965–1.537			
Child-Pugh	1.172	0.323	0.856–1.603			
Tumor size	**1.080**	**<0.001**	**1.046–1.116**			
Tumor number	**1.800**	**<0.001**	**1.494–2.168**	**1.564**	**<0.001**	**1.287–1.901**
MVI	**1.826**	**<0.001**	**1.443–2.311**	**1.562**	**0.001**	**1.285–2.096**
Satellite lesions	**2.550**	**<0.001**	**1.835–3.544**	**1.817**	**0.001**	**1.290–2.560**
Differentiation	**1.253**	**0.044**	**1.006–1.560**			
AFP (>400 ng/ml)	**1.272**	**0.032**	**1.021–1.585**			
TBIL	1.003	0.740	0.986–1.019			
ALT	1.001	0.294	0.999–1.003			
AST	**1.003**	**0.050**	**1.000–1.007**			
CREA	1.004	0.237	0.998–1.010			
INR	1.546	0.435	0.518–4.608			
Fibrinogen	1.093	0.132	0.973–1.228			
PLR (>167.7)	**1.737**	**0.001**	**1.243–2.427**	**1.569**	**0.009**	**1.118–2.202**
NLR (>3.1)	**1.484**	**0.002**	**1.162–1.895**			
AGR (>0.5)	**0.540**	** <0.001**	**0.433–0.674**	**0.696**	**0.002**	**0.551–0.879**

*HR, hazard ratio; CI, confidence interval. The bold indicates statistically significant*.

Similarly, the multivariate analysis for determining prognostic factors of OS was performed (Table 3). AGR (>0.5) was associated with a decreased risk of OS (HR = 0.673; 95% CI: 0.506–0.896; *P* = 0.007). In contrast, tumor size (HR = 1.050; 95% CI: 1.005–1.097; *P* = 0.029), tumor number (HR = 1.502; 95% CI: 1.207–1.871; *P* < 0.001), MVI (HR = 1.772; 95% CI: 1.338–2.347; *P* < 0.001), satellite lesions (HR = 1.857; 95% CI: 1.267–2.722; *P* = 0.002), tumor differentiation (HR = 1.316; 95% CI: 1.013–1.710; *P* = 0.040), and PLR (HR = 1.862; 95% CI: 1.265–2.739; *P* = 0.002) were associated with an increased risk of OS (Table 3).

**Table 3:** Univariate and multivariate analysis of factors related with overall survival in training set.

	**HR**	***P***	**95% CI**	**HR**	***P***	**95% CI**
Age (>60)	1.042	0.780	0.782–1.387			
Gender	1.401	0.088	0.951–2.064			
Positive HBsAg	1.133	0.540	0.759–1.692			
Positive HBeAg	1.370	0.060	0.987–1.901			
Positive HBcAb	0.874	0.744	0.388–1.965			
Liver cirrhosis	1.125	0.395	0.857–1.478			
Child-Pugh	1.042	0.842	0.692–1.569			
Tumor size	**1.118**	**<0.001**	**1.079–1.159**	**1.050**	**0.029**	**1.005–1.097**
Tumor number	**1.724**	**<0.001**	**1.400–2.215**	**1.502**	**<0.001**	**1.207–1.871**
MVI	**2.310**	**<0.001**	**1.774–3.009**	**1.772**	**<0.001**	**1.338–2.347**
Satellite lesions	**2.549**	**<0.001**	**1.770–3.669**	**1.857**	**0.002**	**1.267–2.722**
Differentiation	**1.448**	**0.005**	**1.121–1.871**	**1.316**	**0.040**	**1.013–1.710**
AFP (>400 ng/ml)	**1.453**	**0.004**	**1.125–1.877**			
TBIL	0.988	0.259	0.968–1.009			
ALT	1.001	0.592	0.998–1.003			
AST	**1.005**	**0.004**	**1.002–1.009**			
CREA	1.002	0.538	0.996–1.008			
INR	1.692	0.419	0.473–6.049			
Fibrinogen	**1.233**	**0.002**	**1.081–1.407**			
PLR (>167.7)	**2.243**	**<0.001**	**1.559–3.228**	**1.862**	**0.002**	**1.265–2.739**
NLR (>3.1)	1.573	0.002	1.185–2.087			
AGR (>0.5)	**0.471**	**<0.001**	**0.364–0.609**	**0.673**	**0.007**	**0.506–0.896**

*The bold indicates statistically significant*.

### The Predictive Role of AGR in HCC Patients

**Figure 3:**
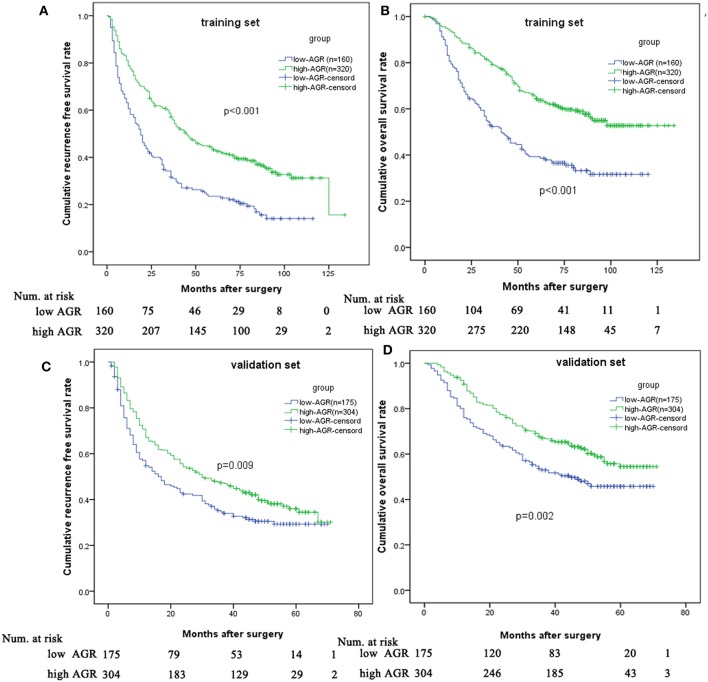
Comparison of prognosis between low and high AGR group in the training set and validation set. Low AGR group had worse RFS **(A)** and OS **(B)** in the training set and validation set **(C,D)** than high AGR group.

In the training set, the 1-, 3-, and 5-year RFS rates were 78.8, 58.7, and 43.2% for the high-AGR group, and 60.6, 31.7, and 23.5% for the low-AGR group, respectively (*p* < 0.001). The 1-, 3-, and 5-year OS rates were 94.7, 79.3, and 64.4% for the high-AGR group, and 83.1, 52.4, and 39.3% for the low-AGR group, respectively (*p* < 0.001). There was statistically significant in the prognosis of HCC patients (Figures 3A,B).

In order to test the predictive value of the AGR for RFS and OS, we further made survival analysis based on the AGR in the validation set. The high-AGR group had a better RFS (1-year RFS: 67.1 vs. 54.7 %; 3-year RFS: 47.3 vs. 34.6 %; 5-year RFS: 36.0 vs. 29.3%, *p* = 0.009). Meanwhile, the high-AGR group had a better OS (1-year survival: 90.8 vs. 76.0 %; 3-year survival: 67.1 vs. 53 %; 5-year survival: 54.4 vs. 45.8% *p* = 0.002) (Figures 3C,D).

Since the albumin in the AGR was affected by liver function, we made the survival analysis in the patients with liver cirrhosis. Interestingly, the high-AGR remained to be associated with better prognosis in term of RFS and OS (Figure S2).

### Comparison of AGR and PLR

As was shown in the multivariate analysis, high PLR as an inflammatory score was inversely correlated with RFS and OS (Tables 2, 3). HCC patients with high PLR had better short-term and long-term survival after hepatectomy (Figure S3). We compared the discriminatory capability of two prognostic inflammatory score using time-dependent receiver operating characteristic curve (ROC). The result suggested that the AUROC of was the comparable for OS and RFS between both prognostic inflammatory scores (Figure S4).

### A Proposal of AGR-PLR Score

**Figure 4:**
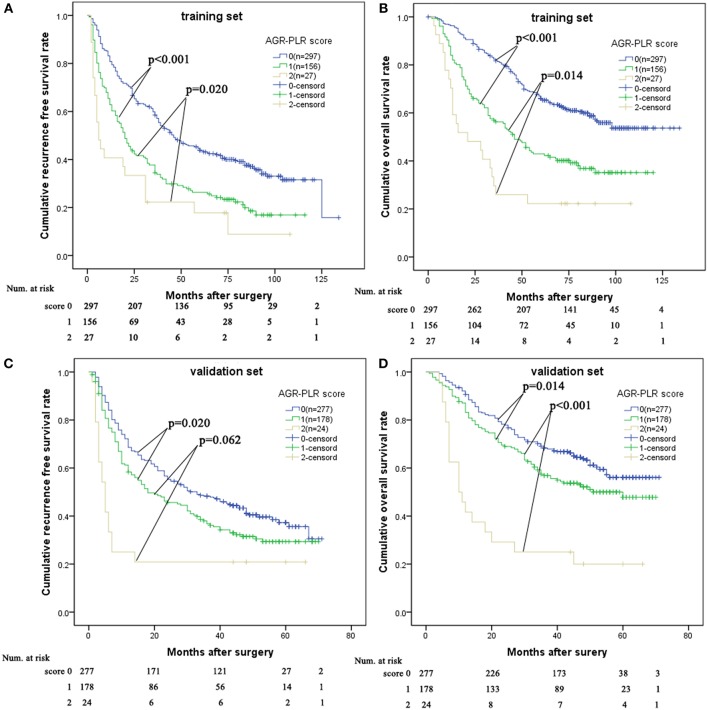
Comparison of prognosis among AGR-PLR score 0, 1, and 2 in the training set and validation set. AGR-PLR score 1 had better RFS and OS than AGR-PLR score 2, worse RFS and OS than AGR-PLR score 0 in the training set **(A,B)** and validation set **(C,D)**.

We allocated 1 point for AGR ≤ 0.5 or PLR > 167.7. The AGR-PLR score consisting of 0, 1, and 2 was built in the training set. There were statistical differences in RFS and OS among the three groups divided according to AGR-PLR score (Figures 4A,B). Patients with AGR-PLR score 1 had better 1-, 3-, and 5-year RFS rates than those with AGR-PLR score 2, but worse than those with AGR-PLR score 0 (score 0:80.8, 58.5, and 43.9% vs. score 1:62.8, 34.5, and 26.3% vs. score 2:40.7, 22.2, and 17.8%, score 0 vs. score 1:*p* < 0.001, score 1 vs. score 2:*p* = 0.062), Similarly, Patients with AGR-PLR score 1 had better 1-, 3-, and 5-year OS rates than those with AGR-PLR score 2, but worse than those with AGR-PLR score 0 (score 0:96.3, 81.7, and 66.0% vs. score 1:84.0, 56.3, and 42.9% vs. score 2:70.4, 25.9, and 22.0%, score 0 vs. score 1:*p* < 0.001, score 1 vs. score 2:*p* = 0.014).

**Figure 5:**
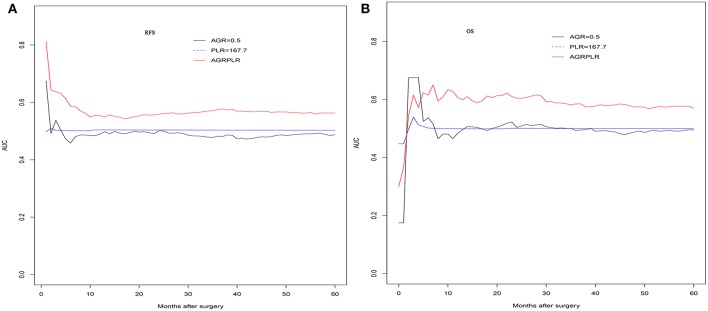
The time-dependent ROC curves of AGR and AGR-PLR in RFS and OS prediction. Compared with AGR, the AUROC of AGR-PLR was the higher for RFS **(A)** and OS **(B)** prediction.

In the validation set, there was also significant significance in term of RFS (AGR-PLR score 0:68.6%%, 48.3%, and 37.3% vs. score 1:58.3, 36.8, and 29.3% vs. score 2:25.0, 20.8, and 20.8%, score 0 vs. score 1: *p* = 0.020, score 1 vs. score 2: *p* = 0.002) and OS (AGR-PLR score 0:90.6%%, 68.3%, and 56.1% vs. score 1:83.1, 56.9, and 47.8% vs. score 2:41.7, 25.0, and 20.0%, score 0 vs. score 1: *p* < 0.001, score 1 vs. score 2: *p* = 0.022) (Figures 4C,D). Based on the result of time-dependent ROC analysis, we found that AGR-PLR score outperformed AGR or PLR in both OS and RFS prediction (Figures 5A,B).

## Discussion

In China, HBV infection was the most common cause of HCC (2). Hepatitis B e Antigen contributes to active inflammatory microenvironment and HCC development (15). For HBV-related HCC patients, positive hepatitis B e Antigen had negative impact on the prognosis (16). Tumor size, tumor number were widely used to define the different stages of HCC, such as by the Barcelona Clinic Liver Cancer (BCLC) and the American Joint Committee on Cancer (AJCC) staging system. They had been investigated as related to the prognosis of HCC after liver transplantation (17), radiofrequency ablation (18), hepatectomy (19), or TACE (20). The presence of MVI and satellite lesion and degree of tumor differentiation can be identified from histological examination. Cumulative evidences suggested that they were the most key pathologically prognostic factors with high predictive power (21–23). Numerous models including these pathologically factors, have been developed specifically to predict tumor recurrence and overall survival with an improvement of prediction power after surgical resection (19, 24). Consistent with above studies, these variables had been investigated as prognostic factors with different hazard ratio in current study.

In recent years, the host inflammatory response caught much attention in tumor progression or metastasis (5). AGR was a novel inflammatory score, which was firstly reported in intrahepatic carcinoma (ICC) (14). Its relationship with the prognosis of HCC after radical therapy remains unclear. As we all known, GGT was expressed in several human neoplasia, and activity of GGT had been widely used for evaluation of active chronic hepatitis and liver fibrosis/cirrhosis (25). The pro-oxidative activity of GGT might contribute to persistent oxidative stress, leading to cell proliferation or apoptosis, and high risk of cancer (26). High serum GGT indicated severe liver inflammation, liver cirrhosis, advanced tumors and adverse outcomes (11). On the other hand, serum albumin level was widely used to assess nutrition status, immune response, and disease progression. Albumin or albumin-based score ALBI had been identified as effective tools to evaluate liver function and predict prognosis (27). High albumin level reflected better nutrition status, liver function and immune response and indicated better outcomes in liver disease (28, 29).

In the current study, we identified that the optimal cutoff value of AGR was 0.5 by using the X-tile software with the strongest discriminatory ability. We found that low AGR (≤0.5), as a reflection of low albumin or high GGT, was related to poor liver function (higher Child-Pugh grade), active underlying inflammation (elevated ALT and AST), high systemic inflammation (higher PLR and NLR), and aggressive tumor characteristics (larger tumor size, multiple tumors, the presence of MVI, and satellite lesion). In order to adjust the effect of other potential risk factors on the AGR, we further adopted the multivariate analysis. We found that AGR > 0.5 was the only favorable factor associated with HCC recurrence and long-term survival in the training set. Based on the survival analysis, patients with AGR> 0.5 had 5-year of 64.4%, while patients with AGR ≤ 0.5 had 5-year of 39.3%. Similarly, in the validation set, we demonstrated that AGR > 0.5 could discriminated a subgroup of HCC patients with better RFS and OS.

We also investigated the role of two other inflammatory score, namely NLR and PLR. The cutoff value of the inflammatory score were 3.1 and 167.7 using the same method. Consistent with other studies (30, 31), the PLR showed prognostic significance in HCC patients. Patients with higher PLR had the worse prognosis. We compared the predictive power of two significant factors in the prognosis of HCCs. The results showed that both the inflammatory scores had the comparable predictive power. In order to improve the prognostic power, we combined the two significant inflammatory scores, namely AGR-PLR score. We made a survival analysis based on the AGR-PLR score in the training set. Interestingly, the AGR-PLR score could be used to stratify individuals in term of RFS and OS. Further, the AGR-PLR score was effectively tested in the validation set. Moreover, the AGR-PLR score outperformed either AGR or PLR in the predicting the prognosis of HCC after hepatectomy. The combination of inflammatory scores might help to better stratify the HCC patients. −2 Log likelihood of the Cox model including all the factors was 2672.563, while −2 Log likelihood of AGR-PLR score was 3733.214. It suggested that the AGR-PLR had no advantages over the Cox model which included all the risk factors. Despite the Cox model might outperform AGR-PLR score, it included more variables and was uneasy to be applied in clinical practice. AGR-PLR score could stratify the HCC patients with distinctive prognosis.

There were some limitations in the current study. Firstly, this was a single-center and retrospective study. In order to reduce the selection bias, we stratified patients into a training set and validation set based on different time spans. Secondly, the presence of major vascular invasion had the strongest negative impacts on the prognosis of HCC patients. It might cover some risk factors. In the current study, we excluded the patients with macro-vascular HCC. Thirdly, some factors differed for OS and RFS in the multivariate analyses because it resulted from the different endpoints. However, the majority of factors affecting the OS and RFS remained the same. Finally, since almost patients included in the study had HBV infection, its prognostic role in HCC with hepatitis C or no infection of hepatitis virus might differ.

In conclusion, we provided strong evidence that the AGR could offer prognostic information on prognosis of patients with HCC after hepatectomy. In addition, we confirmed that AGR-based score (AGR-PLR score) might further stratify the HCC patients with different prognosis.

## Author Contributions

TW proposed the study. JS, LT, and CL performed the research. JS, XZ, and GL collected and analyzed the data. JS wrote the first draft. XZ, WP, TW, and JY reviewed the manuscript. All authors contributed to the interpretation of the study.

### Conflict of Interest Statement

The authors declare that the research was conducted in the absence of any commercial or financial relationships that could be construed as a potential conflict of interest.
