# Supplementary material for: A Novel Index in Hepatocellular Carcinoma Patients After Curative Hepatectomy: Albumin to Gamma-Glutamyltransferase Ratio (AGR)
Source: Front Oncol. 2019 Sep 4;9:817. doi: 10.3389/fonc.2019.00817 (PMC6737307; doi:10.3389/fonc.2019.00817)
Supplement: Supplementary file 1 [file Data_Sheet_1.docx]

**Supplementary table 1**. Patient Clinical Characteristics between low and high AGR group in validation set

|  |  | low AGR group n=175 | high AGR group n=304 | p value |
| --- | --- | --- | --- | --- |
| Age | >60 y | 34(19.4) | 66(21.7) | 0.641 |
| Gender | male | 151(86.3) | 249(81.9) | 0.250 |
| Positive HBsAg |  | 150(85.7) | 265(87.2) | 0.677 |
| Positive HBeAg |  | 46(26.3) | 54(17.8) | 0.035 |
| Positive HBcAb |  | 168(96.0) | 295(97.0) | 0.601 |
| Liver cirrhosis |  | 113(64.6) | 195(64.1) | 1.000 |
| Child-Pugh |  |  |  | 0.001 |
|  | 5 | 151(86.3) | 291(95.7) |  |
|  | 6 | 22(12.6) | 12(3.9) |  |
|  | 7 | 2(1.1) | 1(0.3) |  |
| Tumor size(cm) |  | 8.4±4.8 | 4.9±2.6 | <0.001 |
| Tumor number |  |  |  | 0.411 |
|  | one | 141（80.6） | 253（83.2） |  |
|  | two | 27（15.4） | 35（11.5) |  |
|  | more | 7（4.0） | 16(5.3) |  |
| MVI |  | 64（36.6） | 81（26.6) | 0.023 |
| Satellite lesions |  | 32（18.3） | 43（14.1） | 0.242 |
| Differentiation |  |  |  | 0.179 |
|  | poor | 94(53.7) | 183(60.2) |  |
|  | moderate-well | 81(46.3) | 121(42.2) |  |
| AFP | >400ng/ml | 76(43.4) | 113(37.2) | 0.207 |
| TBIL(umol/L) |  | 17.2±12.8 | 14.1±6.0 | <0.001 |
| ALT(IU/L) |  | 62.0±46.2 | 39.7±26.7 | <0.001 |
| AST(IU/L) |  | 65.1±44.3 | 37.8±24.0 | <0.001 |
| ALB(g/L) |  | 40.3±4.7 | 42.1±3.9 | <0.001 |
| GGT(IU/L) |  | 187.8±203.8 | 41.1±18.8 | <0.001 |
| CREA(umol/L) |  | 75.7±19.6 | 74.1±13.9 | 0.312 |
| PLT(10^9/L) |  | 157.7±87.4 | 138.5±64.8 | 0.012 |
| Neutrophil count | | 3.7±1.6 | 3.3±1.4 | 0.007 |
| Lymphocyte count | | 1.6±0.6 | 1.6±0.6 | 0.945 |
| INR |  | 1.1±0.1 | 1.1±0.1 | <0.001 |
| Fibrinogen |  | 3.1±1.2 | 2.6±0.8 | <0.001 |
| PLR | >167.7 | 24(13.7) | 27(8.9) | 0.123 |
| NLR | >3.1 | 52(29.7) | 58(19.1) | 0.009 |
| Treatment after recurrence | |  |  | 0.040 |
|  | LT | 1(0.6) | 6(2.0) |  |
|  | resection | 5(2.9) | 24(7.9） |  |
|  | Resection+TACE | 3(1.7) | 5（1.6） |  |
|  | RFA | 9(5.1) | 12（3.9） |  |
|  | RFA+TACE | 6(3.4) | 8（2.6） |  |
|  | Resection+RFA | 5(2.9) | 1（0.3） |  |
|  | Resection+RFA+TACE | 1(0.6) | 5（1.6） |  |
|  | TACE+Sorafenib | 0(0) | 2（0.7） |  |
|  | TACE | 49(28.0) | 59（19.4） |  |
|  | Sorafenib | 1(0.6) | 4（1.3） |  |
|  | BSC | 38(21.7) | 59（19.4） |  |

AGR: albumin to gamma-glutamyltransferase ratio, HBsAg: hepatitis B virus surface antigen, HBeAg: hepatitis B virus e antigen, HBcAb: hepatitis B viral core antibody, MVI: microvascular invasion, AFP: alpha-fetoprotein ,TBIL: total bilirubin, ALT: alanine transferase, AST: Aspartate aminotransferase, ALB: albumin, GGT: gamma-glutamyltransferase, CREA: Creatine, PLT: platelet, INR: international normalized ratio, PLR: platelet to lymphocyte ratio; NLR: neutrophil to lymphocyte ratio, LT: liver transplantation, TACE: transarterial chemoembolization, RFA: radiofrequency ablation, BSC: best supportive care.


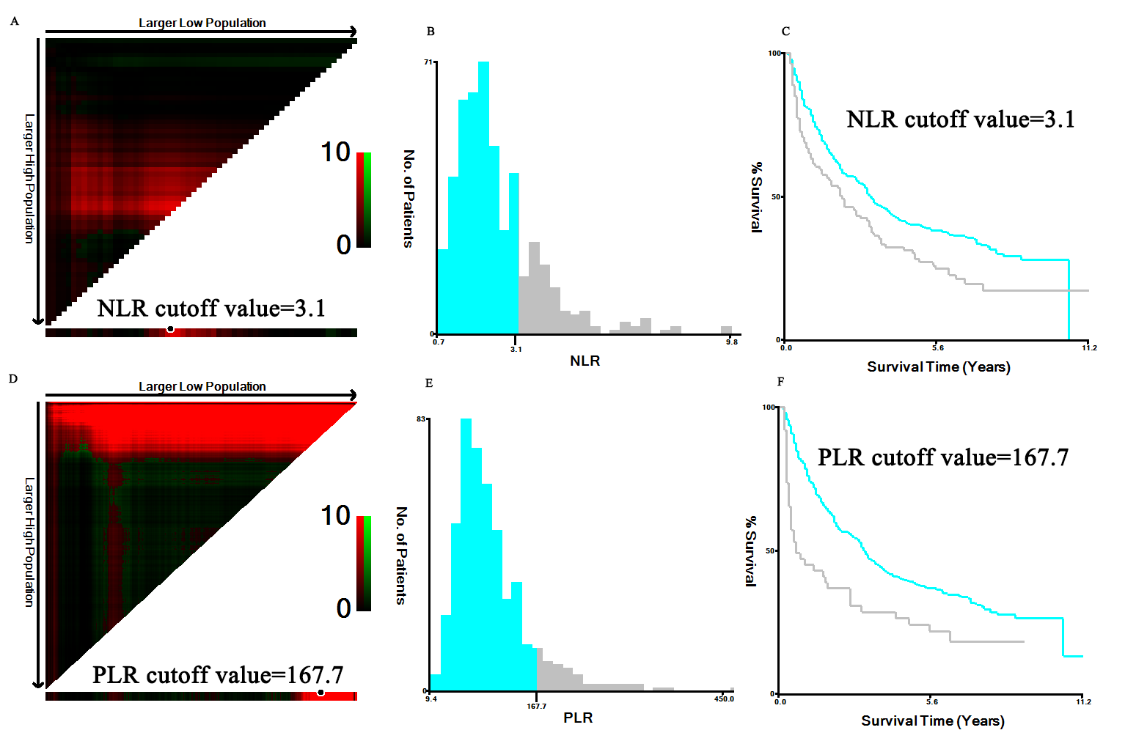


Figure S1. **Identification for the cutoff points produced by X-tile plot in the training set.** The prognostic power was strongest when the cutoff value of PLR and NLR were 167.7 and 3.1, respectively (A, B and C).


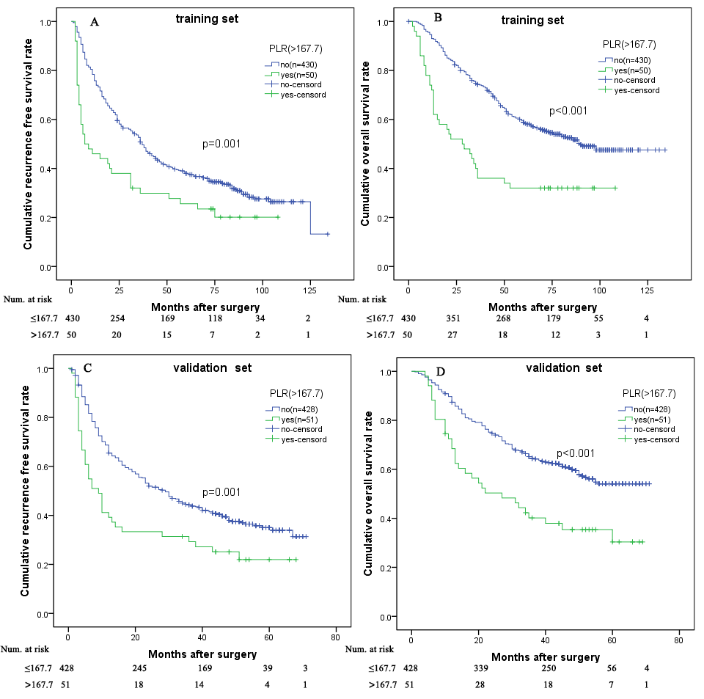


Figure S2. **Comparison of prognosis between low and high PLR group in the training set and validation set.** High PLR group had worse RFS(A) and OS(B) in the training set and validation set (C and D) than low PLR group.


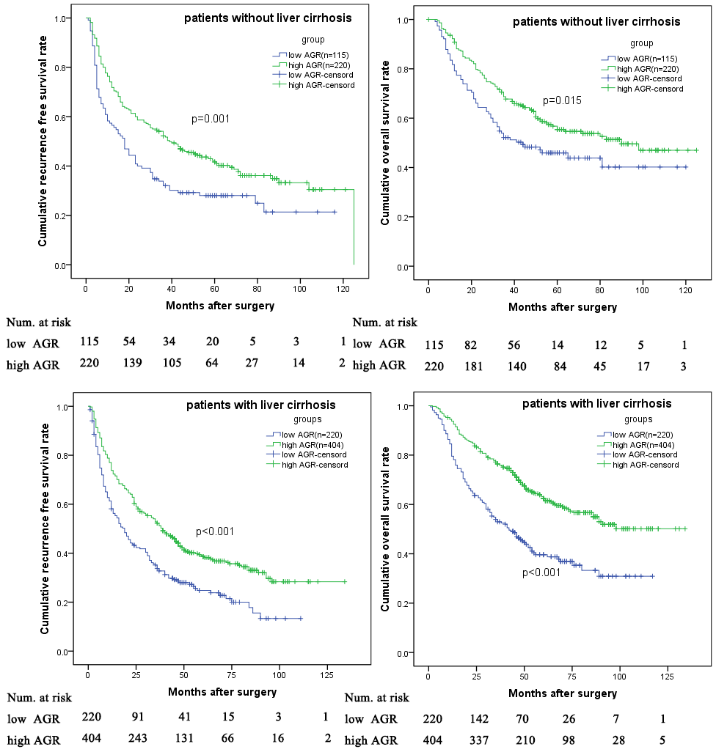


Figure S3. **Comparison of prognosis between low and high AGR group in the patients with liver cirrhosis and patients without liver cirrhosis.** High PLR group had worse RFS(A) and OS(B) among patients without liver cirrhosis and patients without liver cirrhosis (C and D) than low PLR group.


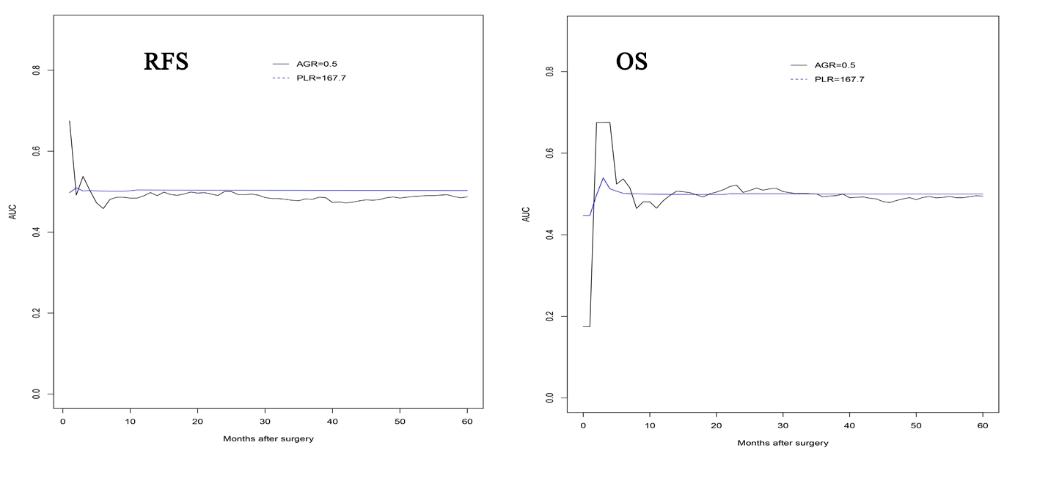


Figure S4. **The time-dependent ROC curves of AGR and PLR in RFS and OS prediction.** Compared with AGR, the AUROC of AGR-PLR was comparable for RFS (A) and OS (B) prediction.
